# Changes in Alcohol Consumption among Users of an Internet Drug Forum during a COVID-19 Lockdown

**DOI:** 10.3390/ijerph192114585

**Published:** 2022-11-07

**Authors:** Bernard Angerville, Marc Moinas, Margaret P. Martinetti, Mickael Naassila, Alain Dervaux

**Affiliations:** 1 Établissement Public de Santé Mentale Barthélémy Durand, 91150 Etampes, France; 2 Groupe de Recherche sur l’Alcool & les Pharmacodépendances (GRAP) INSERM U1247, Université de Picardie Jules Verne, 80054 Amiens, France; 3 Service de Soins de Suite et de Réadaptation en Addictologie, Centre Hospitalier Intercommunal Montdidier Roye, 80700 Roye, France; 4 Department of Psychology, the College of New Jersey, Ewing, NJ 08618, USA; 5 UFR de Médecine, Université Paris-Saclay, 94270 Kremlin-Bicêtre, France; 6 Laboratoire de Recherche PSYCOMADD, Centre Hospitalier Paul Brousse, 94800 Villejuif, France

**Keywords:** alcohol, COVID-19, internet forums, depression, craving

## Abstract

Background: The aim of the present study was to assess the frequency and clinical correlates of users of an Internet drug forum who changed their alcohol use during the March–May 2020 COVID-19 lockdown in France. Methods: An anonymous Internet-based cross-sectional survey during the COVID-19 lockdown was used via messages on a French Internet drug forum. Participants reported any increase in their alcohol consumption during the lockdown. Alcohol craving and depressive/anxiety symptoms were assessed using the Obsessive and Compulsive Drinking scale (OCDS) and Hospital Anxiety and Depression scale (HADS). Results: Of 1310 respondents, 974 (79% of 1270) participants reported alcohol use before lockdown. During the lockdown, 405 participants (41.6%; IC95 (38.5–44.7)) reported an increase. Odds of an increase in alcohol consumption was higher for those with HADS scores higher than 7 (aOR: 2.19; *p* = 0.00002), OCDS scores greater than 7 (aOR: 3.50; *p* < 0.001), and daily psychostimulant use (aOR: 1.85; *p* = 0.002). Conclusions: Users of an Internet drug forum who reported high levels of depressive symptoms, high levels of alcohol craving, and the use of psychostimulants were more likely to increase alcohol consumption during a COVID-19 lockdown.

## 1. Introduction

A mandatory lockdown related to the spread of coronavirus disease 2019 (COVID-19) occurred in France from 17 March to 10 May 2020. The psychological consequences of this lockdown are emerging, both for the general population and for those who use drugs and alcohol [1,2]. Changes in substance use may lead to an increase in health concerns, such as mortality due to overdose [3] and increased alcohol consumption [4]. For example, some authors have found that lockdowns related to the COVID-19 pandemic increased alcohol consumption in the general population [5,6,7]; however, a recent meta-analysis found that 23% of participants reported increases in alcohol consumption and 23% reported a decrease [8]. Two studies reporting on longer term effects of the lockdown found that three years after the 2003 SARS pandemic outbreak, alcohol abuse or dependency symptoms in health-care workers were positively associated with having been quarantined [4]. In a multivariate analysis, after controlling for demographic factors, having been quarantined and having worked in a high-risk location were the two types of exposure significantly associated with these outcomes [4,9]. In a study investigating the change in alcohol consumption among alcohol drinkers from the general French population during the same period, 10.7% reported an increase in their alcohol consumption and 24.4% reported a decrease, while 64.8% indicated no change [10].

The COVID-19 pandemic has led to an inevitable surge in the use of digital technologies due to the social distancing norms and nationwide lockdowns [11,12]. A previous study observed excessive Internet use, notably on forums among Chinese children and adolescents during the outbreak of COVID-19, and that they should be considered as vulnerable individuals during these unprecedented times [13]. A growing number of websites provide a platform for individuals with specific conditions to interact with one another, share experiences, and provide support [14,15]. The use of social networks allowed those who use drugs to discuss, share opinions, and provide advices, especially during the COVID-19 context [16]. However, Internet drug forums are found to express distrust of ‘experts’ and ‘authorities’ who provide official information, whom they perceive as lacking the specialist knowledge that they themselves possess [17]. The user community’s knowledge sharing practices can generate a shared perception of a sufficient or even superior drug use experience and knowledge. Moreover, closure of retail outlets and other drinking venues may had led substances users to use Internet forums to find availability of missing substances [18]. This may lead to overdoses and other risky behavior, and thereby contribute to increased harms related to non-medical use of prescription drugs [19]. Therefore, discussion forums are widely used as a monitoring tool for research purposes [20].

To our knowledge, no study has assessed alcohol consumption changes in Internet drug forum users during the COVID-19 lockdown. The aim of the present study was to assess the frequency and clinical correlates of users of an Internet drug forum who changed their alcohol use during the March–May 2020 COVID-19 lockdown in France.

## 2. Material and Methods

### 2.1. Study Design

An anonymous, Internet-based cross-sectional open survey was conducted between 8 April and 10 May 2020 during the lockdown period in France (17 March to 10 May). Participants were recruited via posts and Internet messages on a French-speaking online forum related to drug use (www.psychoactif.org (accessed on 8 April 2020). This website is a non-governmental, non-medical, and volunteer-led forum dedicated to sharing information on psychoactive drugs and harm reduction measures. At the time of survey administration, this website had 38,500 members. For the current study, the methodology and reporting of the results were based on the Checklist for Reporting Results of Internet E- Surveys (CHERRIES) [21]. The survey took approximately 30 min to complete. The respondents were informed that the process was completely anonymous. The informed consent form contained a plain language description of the study, the approximate duration of the interview, and the ethics committee approval. No incentives were offered to provide results.

### 2.2. Participants

We included voluntary participants who reported using at least one of the following substances: alcohol, cannabis, tobacco, psychostimulants (amphetamine, cocaine…), or opioids. Only French citizens living in French territories were included. Since 30% of visitors on the www.psychoactive.org (accessed on 2 February 2020) web site are living in other francophone countries, we excluded them, due to different lockdown conditions between countries.

Exclusion criteria were an age under 18 and/or prior completion of the survey. For this study, the French National Data processing and Liberties Commission-CNIL was consulted, and our study was in line with French regulations on health research (MR-004 study type of French legislation). The study was registered under the following number: 2,211,053 v 0. No ethical committee was mandatory due to the anonymization process for each participant in the present study. This research was performed in accordance with the ethical standards described in the 1964 Declaration of Helsinki and its later amendments. No advertises were used to promote the survey. No remuneration was provided for participation. No Mechanical Turk (MTurk) online (i.e., Amazon Mechanical Turk) crowdsourcing platform was used.

A total of 3467 people visited the first survey page. There were 1087 people who refused to participate after reading the consent form, the participation rate was 68.6%. In addition, 1070 people did not finish the survey, the completion rate was 44.9%. There were 1310 respondents who completely finished the survey. Only completed questionnaires were analyzed.

### 2.3. Study Variables

All study variables were collected using an Internet-based questionnaire. The questionnaire was written in French and was not available in other languages. Information was collected from all respondents to the survey, via fill-in forms that were completed in the database. A password enabled the clinician in charge of the data treatment to access the database. A pilot study was initially implemented on a group of 10 participants in order to test the feasibility and the good understanding of the questionnaire. They were no randomization of item or questionnaires. The questionnaire included 80 items divided into eight sections and each items provided a non-response option. It was not possible for the participant to review or change their answers. Our settings did not allow to record IP addresses of used cookies in order to identify duplicate records.

- Socio demographic charact and lockdown conditions:
  Declarative data from the survey questions included gender, age, professional status, marital status (married/domestic partnership or single), number of days in lockdown, and lockdown domestic conditions (“How many people are living in your place including you?”, “How many children under 18 years old are living with you during the lockdown?”).
- Alcohol consumption:
  Participants were asked questions pertaining to the consumption of alcohol (“Since the beginning of the lockdown, have you increased, diminished, quit, or maintained your alcohol consumption”). We distinguished three groups of participants: (1) a group of participants who increased alcohol consumption, (2) a group of participants who diminished alcohol consumption, or (3) a group of participants who quit alcohol consumption. These three categories were compared to the group of participants who maintained their alcohol consumption throughout the lockdown. Past 12 months alcohol use was examined using the Alcohol Use Disorder Identification Test (AUDIT). We classified participants of either gender as hazardous drinkers if AUDIT scores were 8 [22].
- Alcohol craving:
  Alcohol craving during lockdown was assessed using Obsessive Compulsive Drinking Scale (OCDS) [23], where higher scores indicate greater craving. Although the OCDS produces a continuous-variable score, we created a dichotomous variable of high or low craving, based on the interquartile range (IQR) of OCDS scores for our sample (above or below the IQR, respectively).
- Tobacco and drugs use:
  Participants were asked questions pertaining to the use of daily tobacco use, cannabis use, psychostimulants (cocaine, crack, amphetamines, ecstasy…) use, heroin use, and daily hallucinogen (LSD, mushrooms) use (“Before the lockdown, did you daily use …?”)
- Depressive and anxiety symptoms during lockdown were assessed using the Hospital Depression and Anxiety scale (HADS) [24]. The HADS is the most used self-reported tool for assessing depression and anxiety symptoms in several populations including those who use substances. Scores higher than 7 for HADS-A or HADS-D are associated with the presence of anxiety or depression symptoms, respectively [25].

### 2.4. Statistical Analysis

Quantitative data were described using mean ± standard deviation (SD) or frequencies and percentages. Proportions of users were presented with 95% confidence intervals (CI). Changes in drinking patterns (increase, decrease, or quit) were considered as qualitative variables (yes or no). The prevalence rates of changes in drinking patterns were correspondingly reported and the Chi-square test (χ^2^) was utilized to compare between-group differences. Univariate/multivariate logistic regression models were further used to explore the potential predictors of changes in alcohol consumption. Variables significantly associated with each drinking pattern in the univariate analysis (i.e., *p*-values less than 0.20) were retained for the multivariate analysis. A backward step by step logistic regression was then performed to identify factors independently associated with changes in drinking patterns. In addition to the variables that were statistically significant in our study, other variables usually reported in the literature, such as age and gender, were also included in the model as adjustment variables, even if they were not statistically significant in our sample. Odds ratios and corresponding 95% CI were estimated using this logistic regression. No statistical correction was necessary to adjust for the non-representative sample. Statistical significance was defined as *p* < 0.05. Results were analyzed with SPSS for Windows 22.0 (IBM, Armonk, NY, USA).

## 3. Results

### 3.1. Sociodemographic and Clinical Characteristics

Demographic data and clinical features are summarized in Table 1. Of 2380 respondents who started the survey, 1310 completed the survey. A total of 974 (79% of 1270) participants reported daily alcohol consumption, of whom 711 (72.9%, IC95 (70.2–75.8)) reported daily tobacco smoking and 718 (73.7%, IC95 (70.9–76.5)) reported daily cannabis use (Table 1). Participants had been under lockdown at home for a mean (SD) of 31.8 ± 7.5 days when they completed this survey, and most participants were male (Table 1).

Regarding the frequency of alcohol consumption before the lockdown, according to the AUDIT, 169 (17.3% IC95 (14.9–19.7)) 4 or more times a week; 298 (30.6% IC95 (27.7–33.5)) 2 to 3 times a week; 369 (37.8% IC95 (34.8–40.9)) 2 to 4 times a month; and 128 (14.1% IC95 (11.2–17.0)) participants were drinking alcohol monthly or less. Before the lockdown, 351 (36.0% IC95 (33.0–39.0)) participants reported drinking 1or 2 standard drinks, containing 10 g of alcohol, on a typical day; 291 (29.9% IC95 (27.0–32.7)) 3 or 4 drinks; 178 (18.3% IC95 (15.0–20.7)) 5 or 6 drinks; 91 (9.3% IC95 (7.5–11.2)) 7, 8, or 9 drinks; 63 (6.5% IC95 (4.9–8.0)) 10 or more drinks on a typical day before lockdown.

During the lockdown 385 (43.8% IC95 (40.6–47.1)) participants reported drinking 1 or 2 drinks (10 gr of pure alcohol) containing alcohol on a typical day before lockdown; 243 (27.7% IC95 (24.8–30.7)) 3 or 4 drinks; 139 (15.8% IC95 (13.5–18.4)) 5 or 6 drinks; 74 (8.4% IC95 (6.7–10.4)) 7, 8, or 9 drinks; 37 (4.2% IC95 (3.0–5.7)) 10 or more drinks on a typical day.

### 3.2. Changes in Alcohol Consumption

In this study, 218 participants (22.4%; IC95 (19.8–25.0)) reported a decrease in their alcohol consumption, 405 participants (41.6%; IC95 (38.5–44.7)) reported an increase, 96 participants (9.8%; IC95 (7.9–11.7)) reported that they stopped drinking, and 255 participants (26.2%; IC95 (23.4–28.9)) reported that they maintained their alcohol consumption at the same level (Table 2). Participants who reported to increase alcohol consumption were mainly those who reported drinking 1or 2 drinks containing alcohol on a typical day before the lockdown (*n* = 173 (42.72%); χ^2^ = 26.52; *p* < 0.001).

Changes (increase, decrease, or quitting) in alcohol consumption were significantly correlated with age, marital status, being alone at home, the presence of children at home, pursuing working activities, professional status, HADS-D scores, HADS-A scores, AUDIT scores, OCDS scores, and psychostimulant use (Table 2).

### 3.3. Logistic Regression Models for Predictors of Increased, Decreased, and Quitting Alcohol Consumption

The predictors of increases in alcohol consumption are shown in Table 3. In this regard, the multivariate logistic regression analyses indicated that increased alcohol consumption was significantly correlated with HADS-D scores > 7 (aOR: 2.19, IC95 (1.44–3.31); *p* = 0.00002), OCDS scores > 7 (aOR: 3.50, IC95 (2.21–5.56); *p* < 0.001), and daily psychostimulant use (aOR: 1.85, IC95 (1.24–2.7); *p* = 0.002).

Conversely, factors such as being a student (aOR: 2.75, IC95 (1.23–6.16); *p* = 0.01), being alone at home (aOR: 0.13, IC95 (0.08–0.22); *p* < 0.0001), and continuing professional activity were significantly associated with decreasing alcohol consumption during the lockdown. Finally, factors such as age of 30–40 (OR:1.19, IC95 (0.06–0.58)) and being female were significantly associated with quitting alcohol consumption during the lockdown.

## 4. Discussion

The present study examined the frequency of Internet drug forum users who changed their alcohol consumption during the COVID-19 pandemic-associated lockdown during the spring of 2020, and the demographic and clinical variables associated with these changes. We found that a self-reported increase in alcohol drinking was associated with a high level of alcohol craving, HADS-D scores higher than 7, and daily psychostimulant co-use.

The frequency of Internet drug forum users who increased their alcohol consumption in our study (41.6%) is greater than the prevalence of increase (12% to 36%) found in a recent meta-analysis of 128 studies including 492,235 subjects in the general population from 58 countries [8]. In the general French population during the same period, 10.7% reported an increase in their alcohol consumption and 24.4% reported a decrease, while 64.8% indicated no change [10].

In this context, the increase in alcohol consumption might be considered as a maladaptive coping strategy to manage the psychological distress due to the pandemic [26]. This increase in alcohol consumption could increase the risk for alcohol use disorders, as suggested by Wu et al. in a study after the SARS outbreak [4]. Prevention should take these factors into account on the emergence of alcohol use disorders in those who use Internet drug forums.

In contrast to the prevalence of increased alcohol consumption, we found that 23% of this sample decreased their alcohol consumption. This frequency is similar to the rates of decrease among the general French population [10]. We also found that 9.8% of Internet drug forum users stopped drinking. Decreasing or quitting alcohol consumption during the lockdown could be linked to an availability-affordability mechanism: Participants reported a reduced number of drinking occasions due to closure of retail outlets and other drinking venues [26] and reduced affordability may have been due to growing unemployment and financial insecurity [27]. These findings on quitting prevalence are novel as no previous study has evaluated the prevalence of individuals who had quitted their alcohol consumption during the COVID-19 lockdown.

Regarding characteristics related to the increase of alcohol consumption, we found that depressive symptoms were associated to the increase of alcohol consumption found in our sample of Internet drug forum users. This finding is in line with a large body of research showing that risky alcohol use commonly co-occurs with depression, and that depression may be a contributing factor to the development of alcohol use disorders [28,29]. This relationship is concerning given that alcohol consumption among individuals with depression is associated with increased severity and duration of depressive symptoms [30,31].

The high level of the OCDS craving scores in participants who reported an increase in their alcohol consumption are in line with other studies that have revealed the predictive role of craving on alcohol consumption [32]. However, none of the previous studies examined craving associated with increases in alcohol consumption during a COVID-19 lockdown. Targeting craving as a clinical predictor of alcohol-use increase within a lockdown period could help prevent health consequences in Internet drug forum users.

In the present study, psychostimulant use was associated with an increase in alcohol consumption. Co-use of alcohol and psychostimulants induces specific pharmacological interactions that may underlie particular changes in the functioning of the brain reward circuit [33]. In this regard, those who use psychostimulants have been shown to be more sensitive to the stimulating properties of alcohol [34]. Co-use of alcohol and psychostimulants may be due to potentiated effects on pleasure and euphoria as well as decreased aversive subjective effects of either alcohol or psychostimulants. The association between increased alcohol consumption and psychostimulant use that we observed is consistent with the findings of a German study in which 23.6–29.1% of users of stimulants increased their alcohol consumption [35]. In contrast to the association with psychostimulant use, we did not find any associations between an increase in alcohol consumption and cannabis or daily tobacco use.

As these findings are considered within the broader context of alcohol and other drug use during the lockdown, we acknowledge some limitations. First, the data were self-reported and not assessed with structured diagnostic interviews. Second, the use of cross-sectional data can identify associations, but not causal relationships. Third, online surveys are less accessible to people who do not have any Internet access. In future studies, longitudinal data would be helpful to observe changes over time and the relative impact of lockdowns. For example, Killgore et al. found an increase in mean AUDIT scores during a lockdown period in a sample recruited from the Amazon Mechanical Turn (MTurk) online crowdsourcing platform [36]. Further, recent research has demonstrated robust correspondence between subjective self-report measures and longitudinal quantitative self-report methods [37]. The participants who had responded may not be representative of the users of the psychoACTIF Website. Finally, the survey was limited to French residents, but we contend that the findings cannot be generalized to other countries.

Despite these limitations, the current study provides novel findings in this unique population of Internet drug forum users at an early stage of the pandemic. Focusing on this timeline may help health authorities understand how a relatively small period can influence alcohol drinking patterns. Online discussion forums have become a useful tool to monitor psychoactive drug-use patterns [38]. Self-established drug-related Internet forums are widely used as an important resource for technical and pharmacological knowledges in the absence of evidence-based literature [39]. However, a clear distance between ‘expert’ and user assessments of risk was previously found, whereas online community members seem to abandon traditional methods of determining credibility that are based on authority and hierarchy, in favor of digital tools and new network approaches [17]. Thus, using digital platforms as both screening- and harm- reduction tools could be useful for advancing ecologically valid health policies [40].

## 5. Conclusions

We found a high prevalence of Internet drug forum users who reported an increased in their alcohol consumption during the COVID-19 lockdown, and this finding could inform health policies to prevent the emergence of alcohol use disorders. In addition, we identified clinical variables such as alcohol craving scores, co-use of psychostimulants, and the presence of depressive symptoms related to this increase in alcohol consumption. These associations may guide efforts to prevent health consequences associated with broader pandemic-related public health measures. Internet drug forums represent a useful tool for future research, harm reduction programs, and prevention strategies on alcohol consumption. Finally, the long-term effects of this increase in alcohol consumption during the COVID-19 lockdown should be evaluated.

## Figures and Tables

**Table 1:** Demographics and clinical characteristics of participants reporting alcohol use (*n* = 974).

Characteristics	Participants (*n* = 974)	%	IC95
Gender			
Male	646	66.3	63.3–69.3
Female	328	33.7	30.7–36.4
AGE (years old)			
18–25	347	35.6	32.6–38.6
25–30	143	14.7	12.5–17.0
30–40	220	22.6	20.0–25.2
>40	191	19.6	17.1–22.1
Marital status			
Married/domestic partnership	455	46.7	43.6–49.8
Not alone at home	751	77.1	74.5–79.8
Presence of children at home	287	29.5	26.6–32.3
Professional status			
Unemployed	212	21.7	19.2–24.4
Working	518	53.2	50.1–56.3
Student	244	25.1	22.3–27.7
Continuing professional activity	113	11.6	9.5–13.6
HADS-D scores			
>7	384	39.4	36.3–42.5
<7	590	60.6	57.4–63.5
HAD-A scores			
>7	462	47.4	44.3–50.5
<7	512	52.6	49.5–55.7
AUDIT scores			
≥8	558	57.3	54.2–60.4
<8	416	42.7	39.6–45.8
OCDS scores			
>7	441	45.2	42.1–48.4
<7	533	54.8	51.7–58.0
Daily tobacco smokers	711	73.0	70.2–75.8
People who use cannabis daily	718	73.7	71.0–76.5
People who use psychostimulant daily	369	37.8	34.8–40.9
People who use Heroin daily	33	3.4	2.2–4.5
People who use hallucinogen daily	195	20.0	17.5–22.5

Data are expressed as median (IQR), *n* (%), or *n* of *N* (%), where *N* is the total number of patients with available data.

**Table 2:** Prevalence of increase, decrease, quitting, and maintaining alcohol use based on demographic variables *n* (%).

	Increased Alcohol Use *n* = 405	Decreased Alcohol Use *n* = 218	Quitting Alcohol Use *n* = 95	Maintain Alcohol Use *n* = 255	χ^2^	*p*-Value
Gender						
Male (*n* = 646)	260 (64.2)	144 (66.0)	58 (61.0)	183 (71.8)	5.2	0.2
Female (*n* = 328)	145 (35.8)	74 (34.0)	37 (39.0)	72 (28.2)		
Age (years old)						
18–25 (*n* = 347)	120 (29.6)	110 (50.4)	46 (55.4)	71 (29.1)	59.6	<0.0001
25–30 (*n* = 143)	85 (21.0)	28 (12.8)	12 (14.5)	47 (19.3)		
30–40 (*n* = 220)	111 (27.4)	37 (17)	8 (9.6)	64 (26.2)		
>40 (*n* = 191)	89 (22.0)	23 (10.1)	17 (20.5)	62 (25.4)		
Married/domestic partnership (*n* = 455)	231 (55.0)	80 (36.7)	24 (25.3)	127 (49.8)	37.6	<0.0001
Not alone at home (*n* = 751)	328 (81.0)	171 (78.4)	73 (75.8)	179 (70.2)	269	<0.0001
Presence of children at home (*n* = 287)	142 (35.0)	52 (23.8)	29 (30.5)	64 (25.1)	11.8	0.008
Continuing professional activity (*n* = 113)	57 (14.0)	12 (5.5)	9 (9.4)	35 (13.7)	11.9	0.008
Professional status						
Unemployed (*n* = 212)	161 (39.7)	46 (21.0)	13 (13.7)	63 (24.7)	5.2	0.18
working (*n* = 518)	224 (55.3)	87 (40.0)	39 (41.0)	155 (60.8)		
Student (*n* = 244)	20 (5.0)	85 (39.0)	43 (45.3)	37 (14.5)		
HADS-D scores						
<7 (*n* = 590)	216 (53.3)	134 (61.5)	61 (63.2)	179 (70.2)	19.2	0.0002
>7 (*n* = 384)	189 (46.7)	84 (38.5)	34 (36.8)	76 (29.8)		
HADS-A scores						
<7 (*n* = 512)	188 (46.4)	123 (56.4)	60 (63.2)	141 (55.3)	12.0	0.007
>7 (*n* = 462)	217 (53.6)	95 (43.6)	35 (36.8)	114 (44.7)		
AUDIT scores						
<8 (*n* = 416)	106 (26.2)	63 (28.9)	49 (51.6)	91 (35.7)	25.0	<0.0001
≥8 (*n* = 558)	299 (73.8)	155 (71.1)	46 (48.4)	164 (64.3)		
OCDS scores						
<7 (*n* = 533)	171 (42.2)	128 (58.7)	72 (75.8)	162 (63.5)	50.8	<0.0001
>7 (*n* = 441)	234 (57.7)	90 (41.3)	24 (25.2)	93 (36.5)		
Daily tobacco smokers (*n* = 711)	298 (73.5)	165 (75.7)	67 (69.8)	181 (71.0)	1.9	0.6
People who use cannabis daily (*n* = 718)	291 (71.8)	176 (80.7)	67 (69.8)	184 (72.1)	7.4	0.06
People who use psychostimulant daily (*n* = 369)	165 (40.7)	104(47.7)	29(30.2)	71 (27.8)	23.7	<0.0001
People who use Heroin daily (*n* = 33)	18 (4.0)	6 (2.7)	1 (1.0)	0 (0.0)	3.3	0.3
People who use hallucinogen daily (*n* = 195))	85 (21.0)	51 (23.4)	17 (17.7)	42 (16.5)	11.8	0.2

*p* values comparing people changing consumption and maintained consumption are from χ^2^ test. HADS = Hospital, Anxiety, and Depression scale. AUDIT: Alcohol Use Disorders Identification test. OCDS = Obsessive and Compulsive scale.

**Table 3:** Odds ratios (95% confidence interval) from univariate analysis for the association of socio-behavioral and health status variables with increased, decreased, and quitting alcohol use.

	Increased Alcohol Use			Decreased Alcohol Use			Quitting Alcohol Use		
	*n* = 405	Unadjusted OR (95% CI)	*p* Value	*n* = 218	Unadjusted OR (95% CI)	*p* Value	*n* = 95	Unadjusted OR (95% CI)	*p* Value
Gender									
Male	260 (64.2)	1 [Reference]	NA	144 (66.0)	1 [Reference]	NA	58 (61.0)	1 [Reference]	NA
Female	145 (35.8)	1.41 (1.0 to 1.9)	0.04	74 (34.0)	0.7 (0.5 to1.1)	0.2	37 (39.0)	1.6 (0.9 to2.6)	NS
Age (years old)									
18–25	120 (29.6)	1 [Reference]	NA	110 (50.4)	1 [Reference]	NA	46 (55.4)	1 [Reference]	NA
25–30	85 (21.0)	0.7 (0.4 to 1.1)	0.159	28 (12.8)	0.4 (0.2 to 0.7)	0.001	12 (14.5)	0.4 (0.2 to 0.8)	0.01
30–40	111 (27.4)	1.0 (0.7–1.6)	0.905	37 (17.0)	0.37 (0.2 to 0.6)	0.0001	8 (9.6)	0.2 (0.1 to 0.4)	<0.0001
>40	89 (22.0)	0.85 (0.54–1.32)	0.464	23 (10.1)	0.24 (0.1 to 0.4)	<0.0001	17(20.5)	0.4 (0.2 to 0.8)	0.01
Married/domestic partnership	231 (55.0)	1.8 (1.4 to 2.5)	<0.0001	80 (36.7)	1.7(1.2 to 2.4)	0.004	24 (25.3)	0.3(0.2 to 0.5)	<0.0001
Not alone at home	328 (81.0)	1.8 (1.3 to 2.6)	0.001	171 (78.4)	8.6 (5.6 to 13)	<0.0001	72 (75.8)	0.1 (0.1 to 0.2)	<0.0001
Presence of children at home	142 (35.0)	1.6 (1.1 to 2.3)	0.007	52 (23.8)	1.0 (07 to1.6)	0.09	29 (30.5)	1.3 (0.8 to 2.2)	0.3
Ongoing work	57 (14.0)	1.0 (0.6 to 1.6)	0.9	12 (5.5)	0.4 (0.2 to 0.7)	0.004	9 (9.4)	0.6 (0.3 to 1.4)	0.3
Professional status									
Unemployed	161 (39.7)	1 [Reference]	NA	46 (21.0)	1 [Reference]	NA	13 (13.7)	1 [Reference]	NA
Working	224 (55.3)	1.1 (0.7 to 1.5)	0.73	87 (40.0)	0.76 (0.5 to 1.2)	0.26	39 (41.0)	0.77 (0.5 to 1.2)	0.26
Student	20 (5.0)	1.48 (0.9 to 2.5)	0.13	85 (39.0)	3.15 (1.8 to 5.4)	<0.0001	43 (45.3)	3.15 (1.8 to 5.4)	<0.0001
HADS-D scores									
<7	216 (53.3)	1 [Reference]	NA	134 (61.5%)	1 [Reference]	NA	61 (63.2)	1 [Reference]	NA
>7	189 (46.7)	2.1 (1.5 to 2.9)	<0.0001	84 (38.5)	1.5 (1.0 to 2.1)	0.05	34 (36.8)	1.3 (0.8 to 2.2)	0.2
HADS-A scores									
<7	188 (46.4)	1 [Reference]	NA	123 (56.4)	1 [Reference]	NA	60 (63.2)	1 [Reference]	NA
>7	217 (53.6)	1.4 (1.0 to 2.0)	0.03	95 (43.6)	0.9 (0.6 to 1.4)	0.06	35 (36.8)	0.7 (0.5 to 1.2)	0.2
AUDIT scores									
<8	106 (26.2)	1 [Reference]	NA	63 (28.9)	1 [Reference]	NA	49 (51.6)	1 [Reference]	NA
≥8	299 (73.8)	1.6 (1.1 to 2.2)	0.01	155 (71.1)	1.3 (0.9 to 2.0)	0.1	46 (48.4)	0.5 (0.3 to 0.8)	0.009
OCDS scores									
<7	171 (42.3)	1 [Reference]	NA	128 (58.7)	1 [Reference]	NA	71 (%)	1 [Reference]	NA
>7	234 (57.7)	2.4 (1.7 to 3.3)	<0.0001	90 (41.3)	1.2 (0.8 to 1.7)	0.3	24 (%)	0.5 (0.3 to 1.0)	0.04
Daily tobacco smokers	298 (73.5)	1.1 (0.8 to 1.6)	0.5	165 (75.7)	1.3 (0.8 to 1.9)	0.3	67 (69.8)	0.9 (0.6 to 1.6)	0.8
People who use cannabis daily	291 (71.8)	0.9 (0.7 to 1.4)	0.9	176 (80.7)	1.6 (1.0 to 2.5)	0.03	67 (69.8)	0.9 (0.5 to 1.5)	0.6
People who use psychostimulant daily	165 (40.7)	1.7 (1.3 to 2.5)	0.001	104 (47.7)	2.4 (1.6 to 3.4)	<0.0001	29 (30.2)	2.4 (0.7 to 1.9)	0.6
People who use Heroin daily	18 (4.0)	1.3 (0.6 to 2.7)	0.5	6 (2.7)	0.8 (0.3 to 2.5)	0.8	1 (1.0)	0.3 (0.04 to 2.6)	0.3
People who use hallucinogen daily	85 (21.0)	1.3(0.9 to 2.0)	0.15	51 (23.4)	1.5 (1.0 to 2.4)	0.06	17 (17.7)	1.1 (0.6 to 2.0)	0.8

OR, Odds ratio; CI, Confidence Interval; OR odds ratios (95% CI) were derived from the univariate logistic regression.

## Data Availability

The data that support the findings of this study are available from the corresponding author, upon reasonable request.
